# Closed-Loop Task Difficulty Adaptation during Virtual Reality Reach-to-Grasp Training Assisted with an Exoskeleton for Stroke Rehabilitation

**DOI:** 10.3389/fnins.2016.00518

**Published:** 2016-11-15

**Authors:** Florian Grimm, Georgios Naros, Alireza Gharabaghi

**Affiliations:** Division of Functional and Restorative Neurosurgery, and Centre for Integrative Neuroscience, Eberhard Karls University of TuebingenTuebingen, Germany

**Keywords:** robot-assisted rehabilitation, robotic rehabilitation, individualized therapy, hemiparesis, motor recovery, upper-limb assistance, reinforcement learning

## Abstract

Stroke patients with severe motor deficits of the upper extremity may practice rehabilitation exercises with the assistance of a multi-joint exoskeleton. Although this technology enables intensive task-oriented training, it may also lead to slacking when the assistance is too supportive. Preserving the engagement of the patients while providing “assistance-as-needed” during the exercises, therefore remains an ongoing challenge. We applied a commercially available seven degree-of-freedom arm exoskeleton to provide passive gravity compensation during task-oriented training in a virtual environment. During this 4-week pilot study, five severely affected chronic stroke patients performed reach-to-grasp exercises resembling activities of daily living. The subjects received virtual reality feedback from their three-dimensional movements. The level of difficulty for the exercise was adjusted by a performance-dependent real-time adaptation algorithm. The goal of this algorithm was the automated improvement of the range of motion. In the course of 20 training and feedback sessions, this unsupervised adaptive training concept led to a progressive increase of the virtual training space (*p* < 0.001) in accordance with the subjects' abilities. This learning curve was paralleled by a concurrent improvement of real world kinematic parameters, i.e., range of motion (*p* = 0.008), accuracy of movement (*p* = 0.01), and movement velocity (*p* < 0.001). Notably, these kinematic gains were paralleled by motor improvements such as increased elbow movement (*p* = 0.001), grip force (*p* < 0.001), and upper extremity Fugl-Meyer-Assessment score from 14.3 ± 5 to 16.9 ± 6.1 (*p* = 0.026). Combining gravity-compensating assistance with adaptive closed-loop feedback in virtual reality provides customized rehabilitation environments for severely affected stroke patients. This approach may facilitate motor learning by progressively challenging the subject in accordance with the individual capacity for functional restoration. It might be necessary to apply concurrent restorative interventions to translate these improvements into relevant functional gains of severely motor impaired patients in activities of daily living.

## Introduction

Despite their participation in standard rehabilitation programs (Jørgensen et al., 1999; Dobkin, 2005), restoration of arm and hand function for activities of daily living is not achieved in the majority of stroke patients. In the first weeks and months after stroke, a positive relationship between the dose of therapy and clinically meaningful improvements has been demonstrated (Lohse et al., 2014; Pollock et al., 2014). In stroke patients with long-standing (>6 months) upper limb paresis, however, treatment effects were small, with no evidence of a dose-response effect of task-specific training on the functional capacity (Lang et al., 2016). This has implications for the use of assistive technologies such as robot-assisted training during stroke rehabilitation. These devices are usually applied to further increase and standardize the amount of therapy. They have the potential to improve arm/hand function and muscle strength, albeit currently available clinical trials provide on the whole only low-quality evidence (Mehrholz et al., 2015). It has, notably, been suggested that technology-assisted improvements during stroke rehabilitation might at least partially be due to unspecific influences such as increased enthusiasm for novel interventions on the part of both patients and therapists (Kwakkel and Meskers, 2014). In particular, a comparison between robot-assisted training and dose-matched conventional physiotherapy in controlled trials revealed no additional, clinically relevant benefits (Lo et al., 2010; Klamroth-Marganska et al., 2014). This might be related to saturation effects. Alternatively, the active robotic assistance might be too supportive when providing “assistance-as-needed” during the exercises (Chase, 2014). More targeted assistance might therefore be necessary during these rehabilitation exercises to maintain engagement without compromising the patients' motivation; i.e., by providing only as much support as necessary and as little as possible (Grimm and Gharabaghi, 2016). In this context, passive gravity compensation with a multi-joint arm exoskeleton may be a viable alternative to active robotic assistance (Housman et al., 2009; Grimm et al., 2016a). In severely affected patients, performance-dependent, neuromuscular electrical stimulation of individual upper limb muscles integrated in the exoskeleton may increase the range of motion even further (Grimm and Gharabaghi, 2016; Grimm et al., 2016b). These approaches focus on the improvement of motor control, which is defined as the ability to make accurate and precise goal-directed movements without reducing movement speed (Reis et al., 2009; Shmuelof et al., 2012), or using compensatory movements (Kitago et al., 2013, 2015). Functional gains in hemiparetic patients, however, are often achieved by movements that aim to compensate the diminished range of motion of the affected limb (Cirstea and Levin, 2000; Grimm et al., 2016a). Although these compensatory strategies might be efficient in short-term task accomplishment, they may lead to long-term complications such as pain and joint-contracture (Cirstea and Levin, 2007; Grimm et al., 2016a). In this context, providing detailed information about how the movement is carried out, i.e., the quality of the movement, is more likely to recover natural movement patterns and avoid compensatory movements, than to provide information about movement outcome only (Cirstea et al., 2006; Cirstea and Levin, 2007; Grimm et al., 2016a). This feedback, however, needs to be provided implicitly, since explicit information has been shown to disrupt motor learning in stroke patients (Boyd and Winstein, 2004, 2006; Cirstea and Levin, 2007). Information on movement quality has therefore been incorporated as implicit closed-loop feedback in the virtual environment of an exoskeleton-based rehabilitation device (Grimm et al., 2016a). Specifically, the continuous visual feedback of the whole arm kinematics allowed the patients to adjust their movement quality online during each task; an approach closely resembling natural motor learning (Grimm et al., 2016a).

Along these lines, virtual reality and interactive video gaming have emerged as treatment approaches in stroke rehabilitation (Laver et al., 2015). They have been used as an adjunct to conventional care (to increase overall therapy time) or compared with the same dose of conventional therapy. These studies have demonstrated benefits in improving upper limb function and activities of daily living, albeit currently available clinical trials tend to provide only low-quality evidence (Laver et al., 2015). Most of these studies were conducted with mildly to moderately affected patients. In the remaining patient group with moderate to severe upper limp impairment, the intervention effects were more heterogeneous and affected by the impairment level, with either no or only modest additional gains in comparison to dose-matched conventional treatments (Housman et al., 2009; Byl et al., 2013; Subramanian et al., 2013).

With respect to the restoration of arm and hand function in severely affected stroke patients in particular, there is still a lack of evidence for additional benefits from technology-assisted interventions for activities of daily living. The only means of providing such evidence is by sufficiently powered, randomized and adequately controlled trials (RCT).

However, such high-quality RCT studies require considerable resources. Pilot data acquired earlier in the course of feasibility studies may provide the rationale and justification for later large-scale RCT. Such studies therefore need to demonstrate significant improvements, with functional relevance for the participating patients. Then again, costly RCT can be avoided when innovative interventions prove to be feasible but not effective with regard to the treatment goal, i.e., that they do not result in functionally relevant upper extremity improvements in severely affected stroke patients.

One recent pilot study, for example, applied brain signals to control an active robotic exoskeleton within the framework of a brain-robot interface (BRI) for stroke rehabilitation. This device provided patient control over the training device via motor imagery-related oscillations of the ipsilesional cortex (Brauchle et al., 2015). The study illustrated that a BRI may successfully link three-dimensional robotic training to the participant's effort. Furthermore, the BRI allowed the severely impaired stroke patients to perform task-oriented activities with a physiologically controlled multi-joint exoskeleton. However, this approach did not result in significant upper limb improvements with functional relevance for the participating patients. This training approach was potentially too challenging and may even have frustrated the patients (Fels et al., 2015). The patients' cognitive resources for coping with the mental load of performing such a neurofeedback task must therefore be taken into consideration (Bauer and Gharabaghi, 2015a; Naros and Gharabaghi, 2015). Mathematical modeling on the basis of Bayesian simulation indicates that this might be achieved when the task difficulty is adapted in the course of the training (Bauer and Gharabaghi, 2015b). Such an adaptation strategy has the potential to facilitate reinforcement learning (Naros et al., 2016b) by progressively challenging the patient (Naros and Gharabaghi, 2015). Recent studies explored automated adaptation of training difficulty in stroke rehabilitation of less severely affected patients (Metzger et al., 2014; Wittmann et al., 2015). More specifically, both robot-assisted rehabilitation of proprioceptive hand function (Metzger et al., 2014) and inertial sensor-based virtual reality feedback of the arm (Wittmann et al., 2015) benefit from assessment-driven adjustments of exercise difficulty. Furthermore, a direct comparison between adaptive BRI training and non-adaptive training (Naros et al., 2016b) or sham adaptation (Bauer et al., 2016a) in healthy patients revealed the impact of reinforcement-based adaptation for the improvement of performance. Moreover, the exercise difficulty has been shown to influence the learning incentive during the training; more specifically, the optimal difficulty level could be determined empirically while disentangling the relative contribution of neurofeedback specificity and sensitivity (Bauer et al., 2016b).

In the present 4-week pilot study, we combined these approaches and customized them for the requirements of patients with severe upper extremity impairment by applying a multi-joint exoskeleton for task-oriented arm and hand training in an adaptive virtual environment. Notably, due to the severity of their impairment, these patients were not able to practice the reach-to-grasp movements without the exoskeleton. The set-up was, however, limited to pure antigravity support, i.e., it provided passive rather than active assistance. Furthermore, it tested the feasibility of closed-loop online adaptation of exercise difficulty and aimed at automated progression of task challenge.

## Methods

We recruited five stroke patients (2 female, mean age: 52 ± 9 [from 41 to 63] years) in the chronic phase after stroke (65 ± 59 [from 8 to 156] months) who provided written, informed consent and presented with a severe and persistent hemiparesis (for details, see Table 1). The modified upper extremity Fugl-Meyer-Assessment score (i.e., mean motor UE-FMA score without coordination, speed and reflexes) of our group of patients was 14.3 ± 5.3 [from 9 to 22.4]. This study was approved by the ethical review committee of the local medical faculty. It involved a 20-session training program in the course of 4 weeks. Each session consisted of brain self-regulation and proprioceptive feedback with a hand robot (Naros and Gharabaghi, 2015) prior to a physiotherapy training with a multi-joint exoskeleton attached to the impaired arm (Grimm et al., 2016a). Each physiotherapy session consisted of 150 trials of task-oriented reach-to-grasp exercises resembling activities of daily living which were randomly distributed in the directions x, y and z (a total of 50 trials in each direction). The general experimental set-up has already been described in detail elsewhere (Grimm and Gharabaghi, 2016; Grimm et al., 2016a,b) and is cited here when applied in the same way.

**Table 1:** **Clinical information**.

	**Age**	**Sex**	**Months post stroke**	**Side of Insult**	**Type of stroke**	**Affected vessel**	**UE FMA**
Subject 1	63	female	78	right	ischemic	ACM	16.1
Subject 2	52	male	156	right	ischemic	ACI	22.4
Subject 3	59	female	20	left	ischemic	ACM	10
Subject 4	41	male	62	right	ischemic	ACM	9
Subject 5	48	male	8	left	ischemic	ACI	14

ACI, internal carotid artery; ACM, middle cerebral artery.

### Exoskeleton and virtual reality

**Figure 1:**
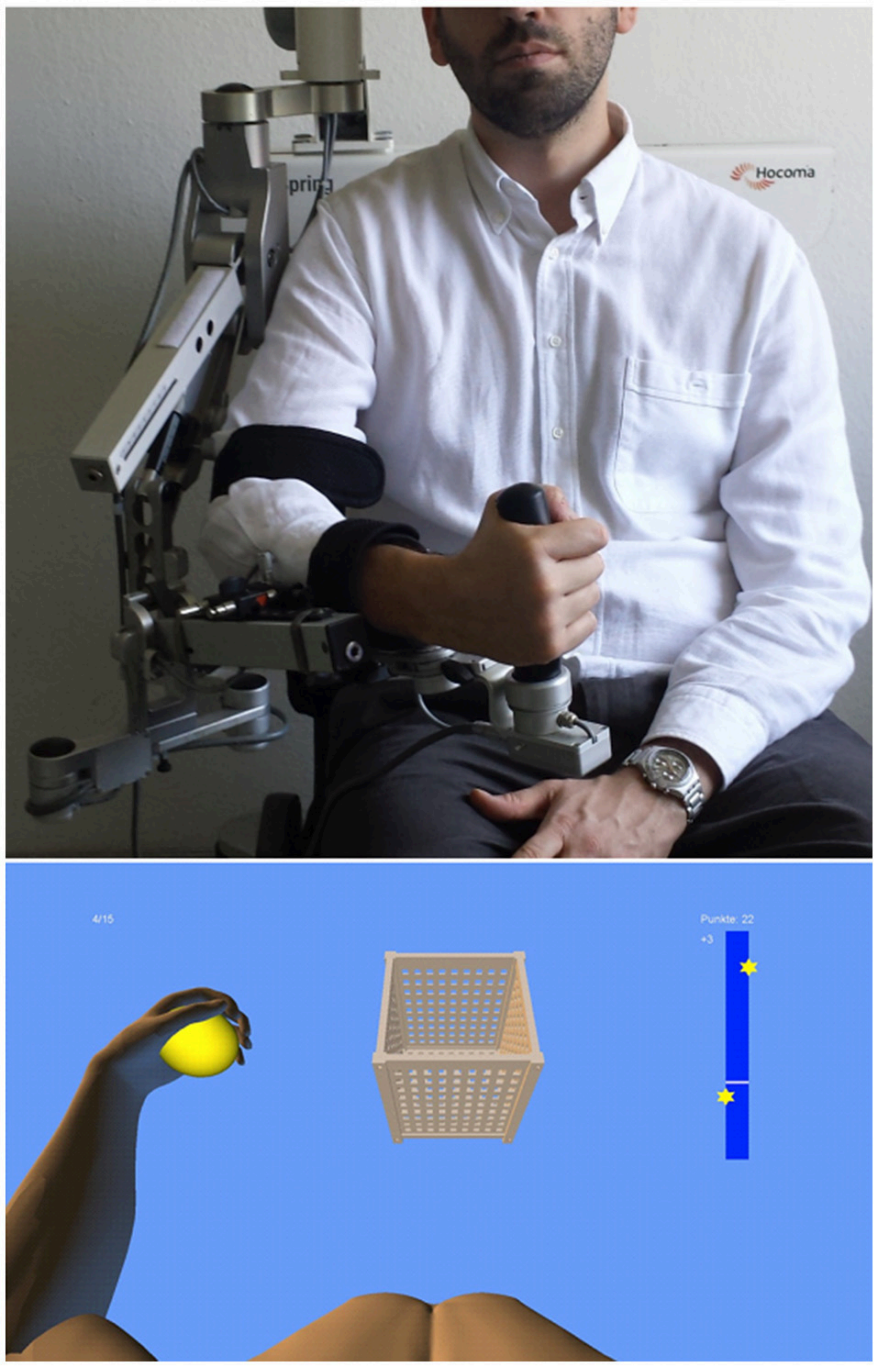
**Training set-up with the exoskeleton (upper row) and the provided visual feedback in virtual reality (lower row)**.

We used a commercially available (Armeo Spring, Hocoma, Volketswil, Switzerland) rehabilitation exoskeleton for shoulder, elbow and wrist joints, with seven axes (i.e., degrees of freedom) providing antigravity support for the paretic arm and registration of movement kinematics and grip force (Figure 1, upper row). This device allowed individual adjustments e.g., of gravity compensation, thereby supporting subjects with severe impairment in performing task-oriented practice within a motivating virtual environment.

We extended these features in-house by using the real-time sensor data of the exoskeleton to display a three-dimensional multi-joint visualization of the user's arm in virtual reality (Figure 1, lower row). This provided feedback as to the movement quality, i.e., the absence or presence of compensatory movements. Such a feedback is more liable to recover movement patterns used by the subject before suffering a stroke. It can also avoid compensatory movements rather than merely providing information about movement execution (Cirstea and Levin, 2007). For this purpose, we used a file mapping communication protocol to capture the angles of all arm joints and the grip force from a shared memory block. The virtual arm engine was programmed in a Microsoft XNA™ framework. The arm model utilized by the engine was constructed as a meshed bone-skin combination with 54 bones (3Ds Max 2010™, Autodesk). The joint angles and grip forces of the device measured with the exoskeleton were used to modify the bone-vectors of the meshed model in accordance with the movements of the user, thus providing online closed-loop feedback. The joint angles of the exoskeleton were directly represented in virtual reality, whereas the grip forces were augmented to feedback natural hand function. More specifically, the maximum grip pressure measured by the force sensor resulted in a full closure of the virtual hand to a fist independent of the subjects' actual ability to perform this particular movement. Prior to each session, subjects were instructed to perform a natural reach-to-grasp movement during the task by using distal (elbow) rather than proximal (shoulder) movements. The three-dimensional visualization of the arm was then applied during each task as an implicit online feedback of the movement, since explicit information can disrupt motor learning in stroke patients (Boyd and Winstein, 2004; Cirstea and Levin, 2007). More specifically, delivery of explicit instructions has been shown to disrupt implicit motor learning after stroke regardless of task (either continuous or discrete movement tasks) or lesion location (involving either the sensorimotor cortical areas or basal ganglia); this disruption did not occur in healthy control subjects (Boyd and Winstein, 2006). In the current set-up, various virtual training paradigms were designed to allow for different rehabilitation exercises resembling activities of daily living.

### Task design

In this study, subjects performed a reach-to-grasp movement toward a ball which changed its position in virtual space after each trial, thus necessitating three-dimensional transfer movements. The ball had to be grasped, carried to a distant basket and then released without the necessity for a final wrist movement. As soon as it entered a defined range around the ball, the virtual hand could react with the former. The ball changed its color according to the hand position (white: out of range, green: possible to grasp, yellow: possible to transfer, red: possible to release). The grasping and releasing of the virtual ball was performed by applying force to the grip sensor and opening the hand, respectively. The respective thresholds of the grip sensor were adjusted to the individual strength of the user.

### Closed-loop adaptation of task difficulty

**Figure 2:**
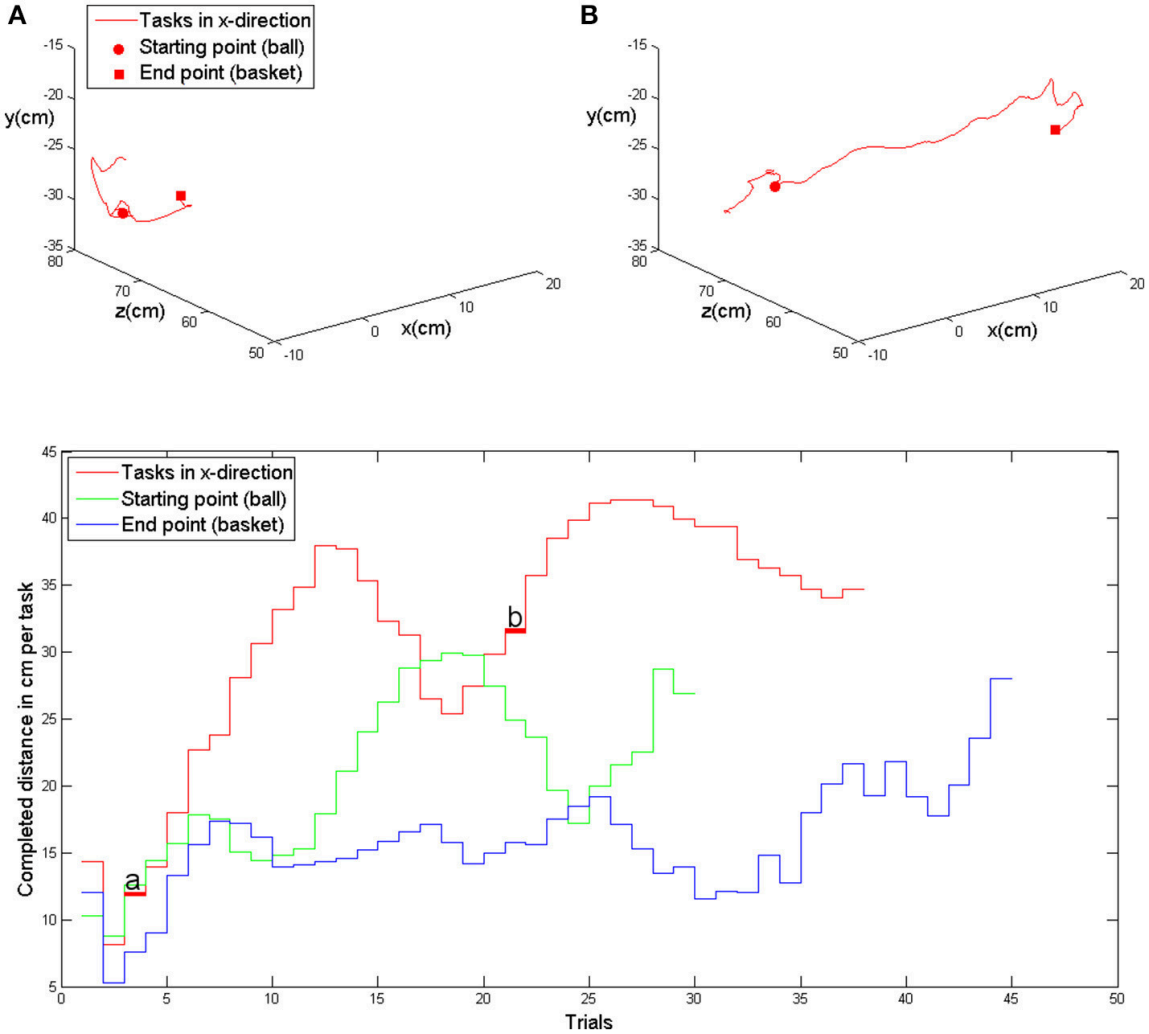
**Upper row: exemplary kinematic data of movement in the x-direction (patient 5, first training session) with the evolution of the task distance in the course of the trials, i.e., at the beginning of the session (A)** and in the middle of the session **(B)**. Lower row: evolution of achieved distances in x-, y- and z-direction in the course of one training session (same as above). The trials shown above are marked with **(A)** and **(B)**.

Modification of task difficulty was achieved by adjusting the virtual training space, i.e., the distance between the ball and the basket, in the course of one session, and from session to session. More specifically, during the device calibration, an individual base point was estimated for every subject at the beginning of the training and remained stable throughout the sessions. This base point was projected in the middle of the sagittal body axis in front of the subject, serving as a reference for symmetrical transfer movements in x (right-left), y (up-down) and z (front-back) direction. The basket and ball were randomly distributed in the virtual space, allowing for 6 movement directions (right, left, up, down, forward, backward). The distances reached during each task were recorded throughout the training and gradually enlarged by the training algorithm. The starting distance between ball and basket was set at 5 virtual units in x, y or z direction (vu), corresponding to 7 cm. Upon successful completion of the task, which was not limited in time, the next task was immediately presented. Whenever the task was successfully accomplished twice, an auto-adaptive algorithm progressively enlarged this distance. In this case, the distance between the objects was enlarged by 7 cm in the corresponding direction. The reached distances were stored at the end of each session and provided the starting distances for the next training day. If the task could not be accomplished, i.e., if one object (ball or basket) could not be reached, the distance was reduced again. To allow for enough time to complete the movement, a timeout of 2 s was given. Following this period, the object moved slowly toward the virtual hand at a velocity of 0.5 vu/s until it could be reached. The new, reduced distance was stored for the next task. Similarly, the grip force required for initiating the augmented closing and opening of the fingers of the virtual model was also progressively increased whenever the respective threshold was achieved three times in a row, and decreased when the necessary force could not be applied. These performance-dependent adjustments enabled the subjects to complete the tasks at their respective capability levels. The subjects were instructed to perform the tasks as quickly and as accurately as possible. To maintain their motivation, they received additional feedback via a point score system: the larger the accomplished distance and the faster the performance, the higher the score per trial. In addition, the total score and the five highest trial scores were displayed to the subjects at the end of each session (Figure 2).

### Outcome measures

The training space of the exoskeleton (real space) and the virtual space correspond linearly with an arbitrary point O (0/0/0) localized in the center of the shoulder joint. All quantitative data are transformed to SI-Units. Since no direct conversion is available, raw sensor data are displayed for the grip force. The kinematic assessment included accuracy, temporal efficiency and range of motion (volume). Movement accuracy was captured by calculating changes of movement direction along an optimal path toward the targets, by estimating the distance function between the hand-position and the final endpoint, and by calculating the second derivative of the function to acquire the number of turning points for each task (Cirstea et al., 2006). Temporal efficiency was captured as the time required to complete each task, and as the mean and peak velocity of the hand between the targets while calculating their distance for x-, y-, and z-directions in virtual units (vu). The range of motion (volume) was measured according to the orthosis and displayed in degrees. The range of sensor-data from the grip-sensor was estimated as the mean change in grip pressure. Furthermore, the raw movement data of all joints (shoulder, upper-arm, elbow and wrist) was acquired in degrees. Movements were allowed in 3D space, i.e., moving simultaneously in x-, y-, and z-direction, as illustrated in Figure 2. However, the outcome measure “mean distance” refers to an arithmetic mean, since the targets were aligned in one axis (x, y, or z) for each task. The average distance covered in the corresponding direction thus reflected the increase of the inter-target distance. Providing the distance in 3D space would have provided (particularly in the first sessions) false positive values due to large inaccuracies during movement execution. As a cumulative parameter of the performance evolution in 3D space we computed the total training volume, which grew along with the subjects' abilities. This volume was estimated on the basis of the performed movement in 3D space (not on the basis of the inter-target distance).

### Statistics

Statistical analysis was performed on a Matlab (2010b) Engine. The kinematic data (volume, distance, grip pressure and joint movement) was tested for linear distribution using the Lilliefors-test (2-sided goodness-of-fit test). The non-parametric Kruskal-Wallis was used for group comparisons of the UE-FMA score between pre- and post-training. To estimate the evolution of parameters during training, a robust multilinear regression model was fitted. Since the Lilliefors-test revealed normality of the data, a robust multilinear regression analysis was applied to minimize the impact of outliers (Holland and Welsch, 1977). The fitting function was based on an iteratively reweighted least squares algorithm. The weight of each iteration was calculated by applying a bi-squared function to the residuals of the previous iteration. The slope b of coefficient estimates and the ratio of the standard error of coefficient estimates (t) are presented for every fitting function. The significance level was set to *p* = 0.05 for all tests.

## Results

All subjects were able to perform the reach-to-grasp exercises in virtual space due to gravity compensation and alteration of the grip force of the exoskeleton.

**Figure 3:**
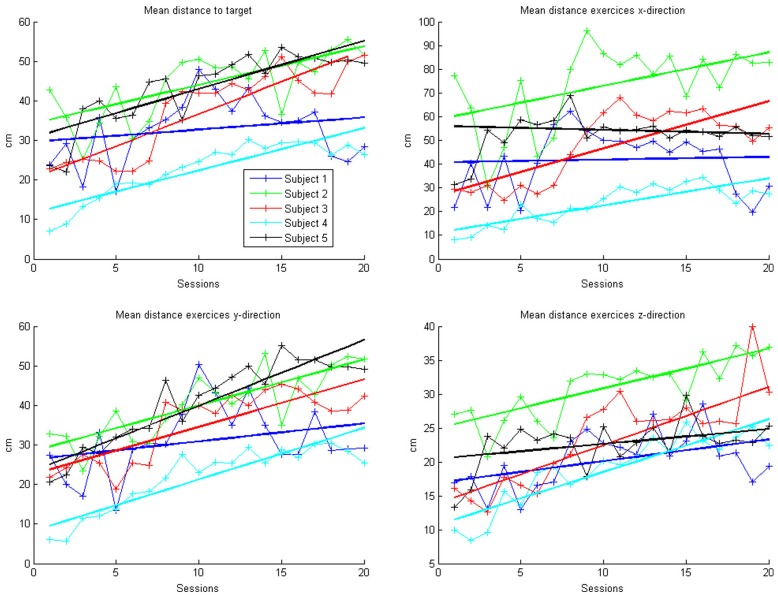
**Evolution of mean arithmetic distance of all directions together and distances for x-, y- and z-directions in the course of the training for each patient**. Each point represents the mean across 50 trials in each direction for each subject. The color indicates the different patients. One session was performed per day. The solid lines indicate the linear regressions in the course of training.

**Figure 4:**
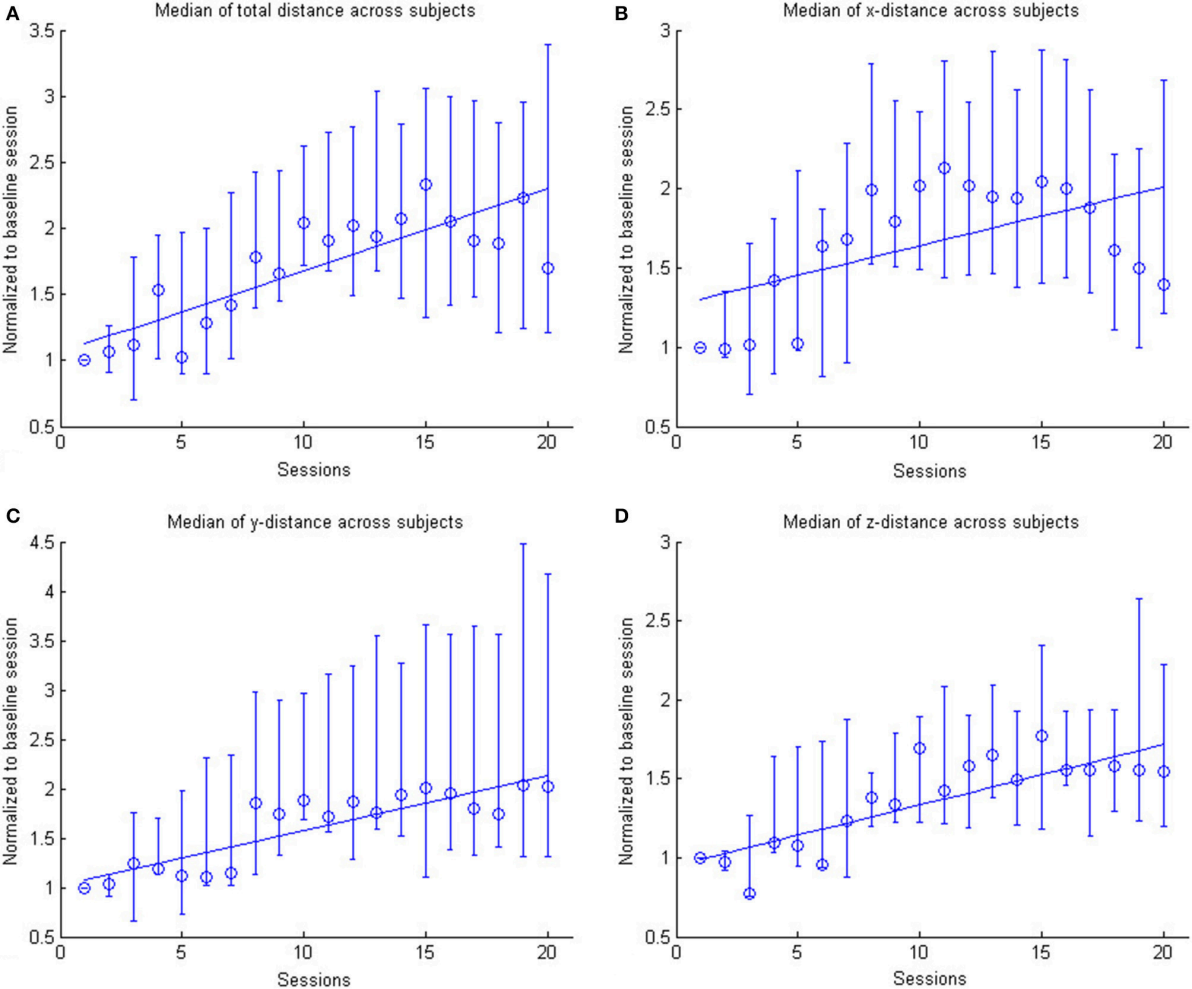
**The figure shows the across subject evolution of the distance traveled in the x, y, and z directions**. The median distance is estimated across subjects, where the distance is the average distance covered in the corresponding direction across trials per session per subject. The data is normalized to the baseline session. Represented are the median group values (dots), the 95% confidence interval and the linear regression (solid line). The subplots shows the evolution of the **(A)** arithmetic mean distance, **(B)** the distance in the x- direction, **(C)** the distance in the y-direction and **(D)** the distance in the z-direction.

**Figure 5:**
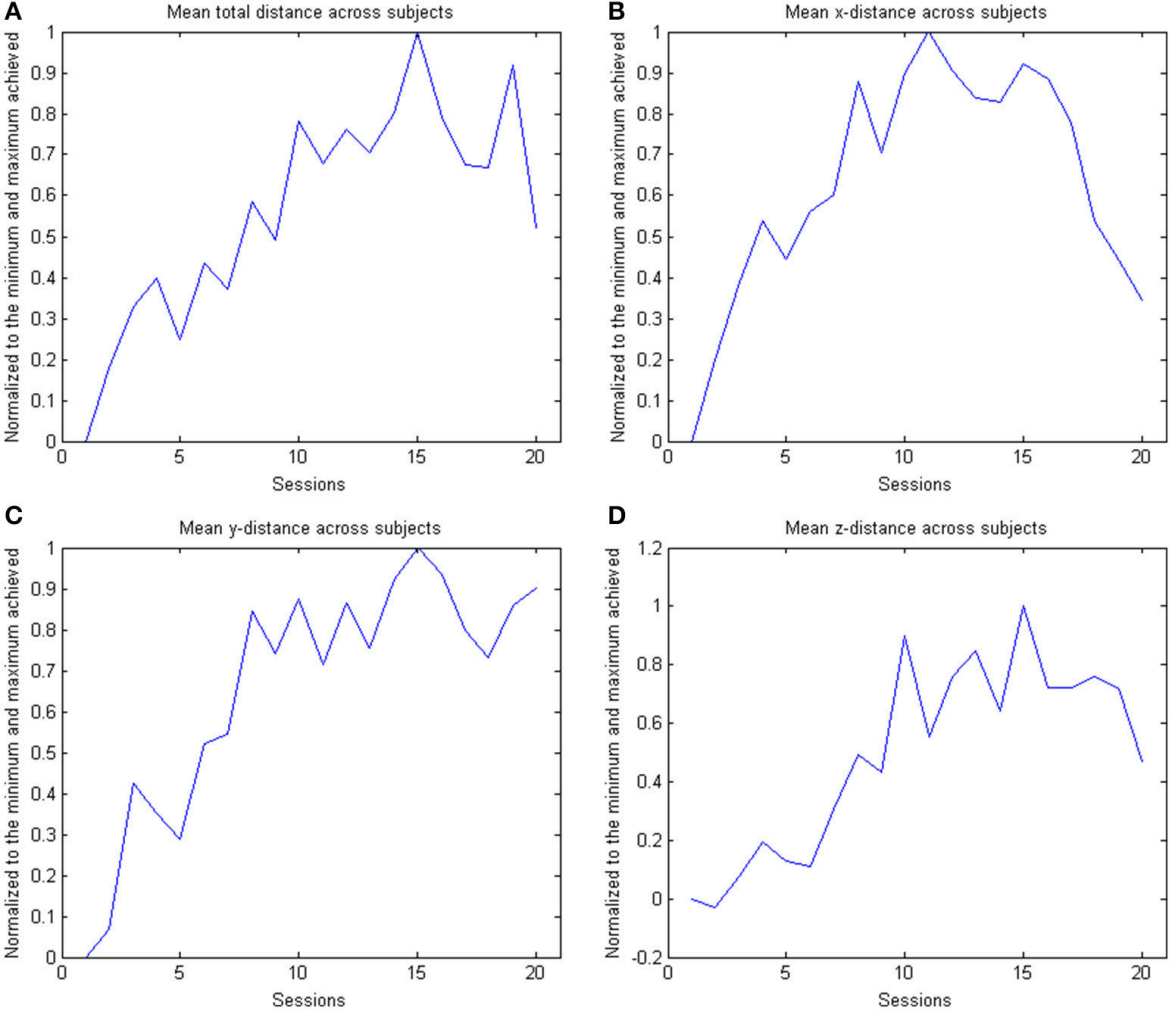
**The figure shows the across subject evolution of the distance traveled in the x, y, and z directions**. The mean distance is estimated across subjects, where the distance is the average distance covered in the corresponding direction across trials per session per subject. The data is normalized to the maximum performance achieved in each parameter. The subplots shows the evolution of the **(A)** arithmetic mean distance, **(B)** the distance in the x- direction, **(C)** the distance in the y-direction and **(D)** the distance in the z-direction.

In the course of 20 sessions, the auto-adaptive algorithm led to a progressive increase of the training space in accordance with the subjects' abilities (Figure 3: individual subjects, Figure 4: group data normalized to baseline, Figure 5: group data normalized to maximum). The results are presented in Table 2. The gain was particularly high in the first 2–3 sessions, and reached a plateau in the last 3–4 sessions. The mean distance, and the distances for the y-direction and the z-direction in virtual space all showed a significant increase throughout all sessions. The trend in the x-direction (*p* = 0.057) for all sessions reached significance when considering the evolution before the saturation effect, i.e., sessions 1–18.

**Table 2:** **Parameter progression over training**.

	**Mean pre**	**Mean post**	**Linear regression**		
			**b**	**t**	***p***
Mean arithmetic distance in cm	24.0±12.6	31.2±20.9	0.058	6.4	<0.001
Distance x-direction in cm	33.6±26.1	49.6±22.1	0.05	5.1	<0.001
Distance y-direction in cm	21.7±10.0	39.5±11.8	0.049	5.9	<0.001
Distance z-direction in cm	16.7±6.4	26.8±6.9	0.042	7.7	<0.001
Volume in cm^3^	18054±26053	35572±15069	0.089	4.4	0.008
Inaccuracy in number of errors	13.5±9.4	9.5±5.7	0.004	0.9	0.01
Peak velocity in cm/s	6.9±2.7	8.9±2.6	0.4	6.8	<0.001
Time per task in s	14.9±5.8	7.4±3.0*s*	0.005	1.5	0.01
Elbow movement in °	8.9±3.3	13.7±5.5	0.021	3.0	0.001
Grip force in pu	0.031±0.01	0.069±0.03	0.0023	3.1	0.001
UE-FMA	14.3±5.4	16.9±6.1	−	−	−

This learning curve was paralleled by an improvement of kinematic parameters (Figure 5, Table 2): The mean training volume increased over the time course of training (pre: 18054 cm^3^ ± 26053 cm^3^; post: 35572 cm^3^ ± 15069 cm^3^), reaching a robust average increase of at least 100% of the starting volume from the 6th session on. This improvement was paralleled by a temporary (i.e., sessions 7–18) increase of volume variability, indicating the potential for relevantly larger gains in some of the subjects.

This gain in range of motion was not at the expense of other kinematic parameters. By contrast, both the inaccuracy (number of turning points) and movement speed- related parameters such as peak velocity and time per task also improved. The peak velocity revealed a robust average increase of at least 50% of the starting speed from the 10th session on. The variability also increased steadily, suggesting that subjects have different specific slopes of increased speed.

**Figure 6:**
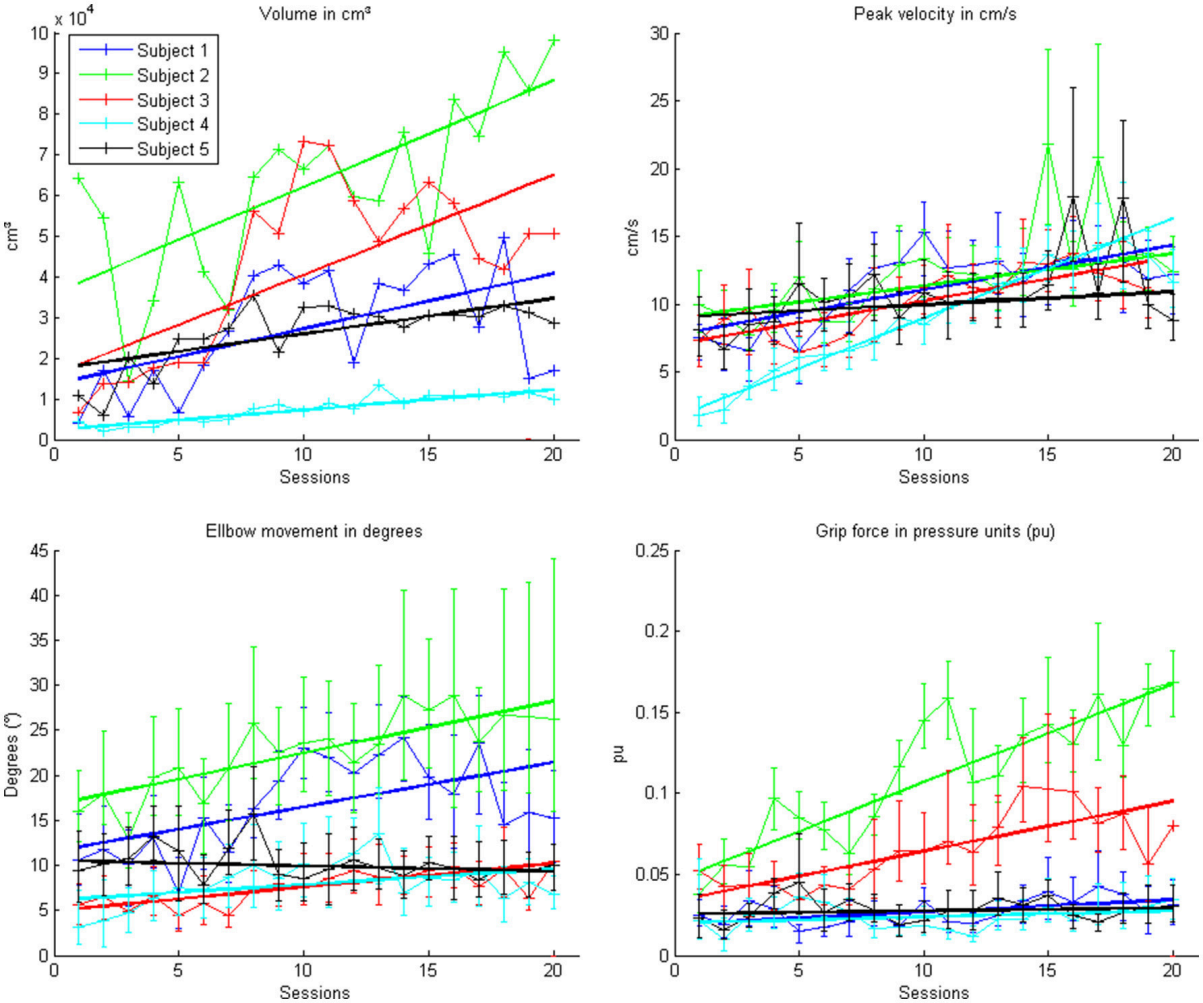
**Evolution of the kinematic parameters volume, peak velocity, elbow movement and mean grip force in the course of the training for each patient**. Each point represents the mean across 150 trials for each patient. The color indicates the different patients. One session was performed per day. The solid lines indicate the linear regressions in the course of training.

**Figure 7:**
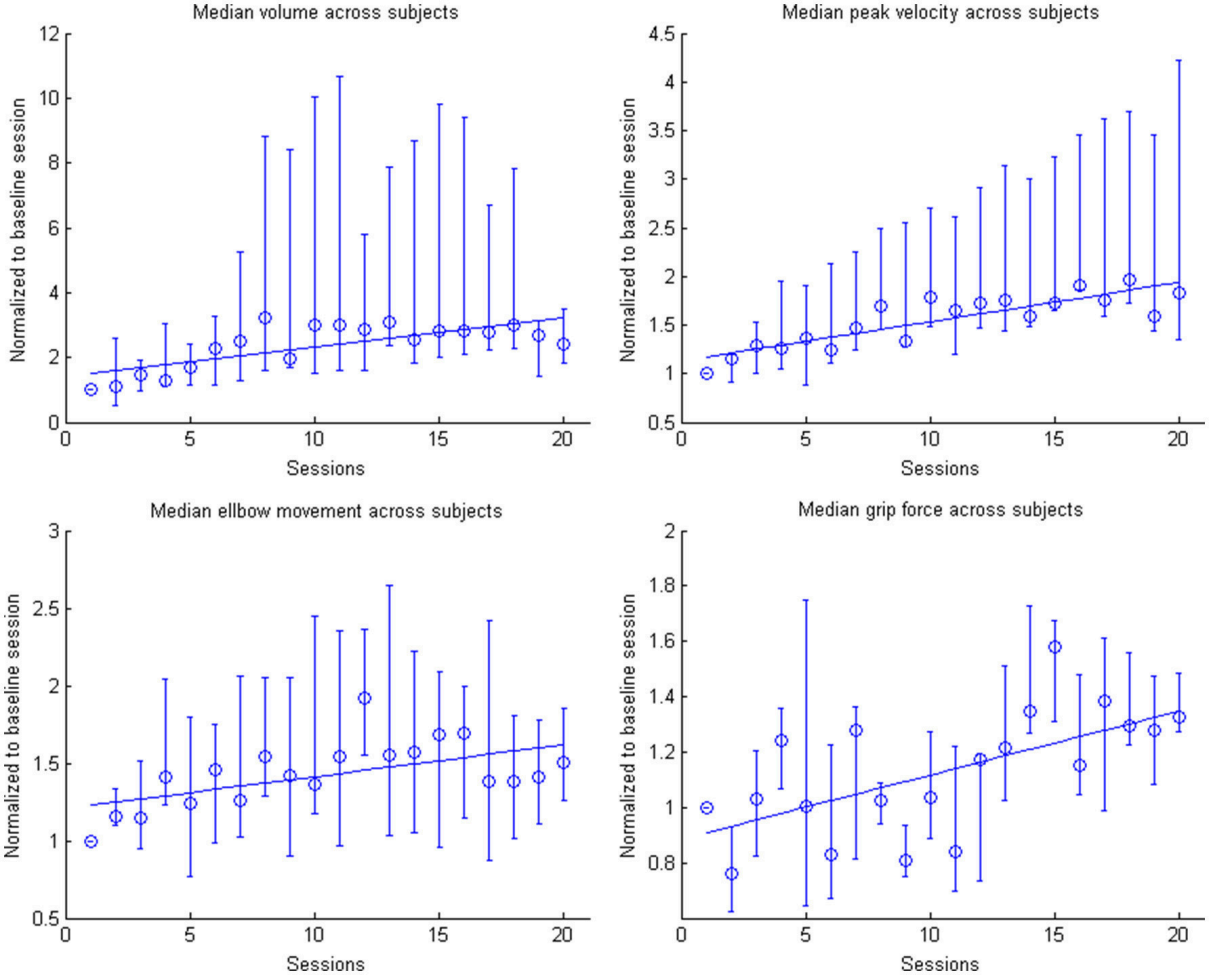
**Evolution of the kinematic parameters volume, peak velocity, elbow movement and mean grip force in the course of the training for the group**. Data is normalized to the first day. Represented are the median group values (dots), the 95% confidence interval and the linear regression (solid line).

**Figure 8:**
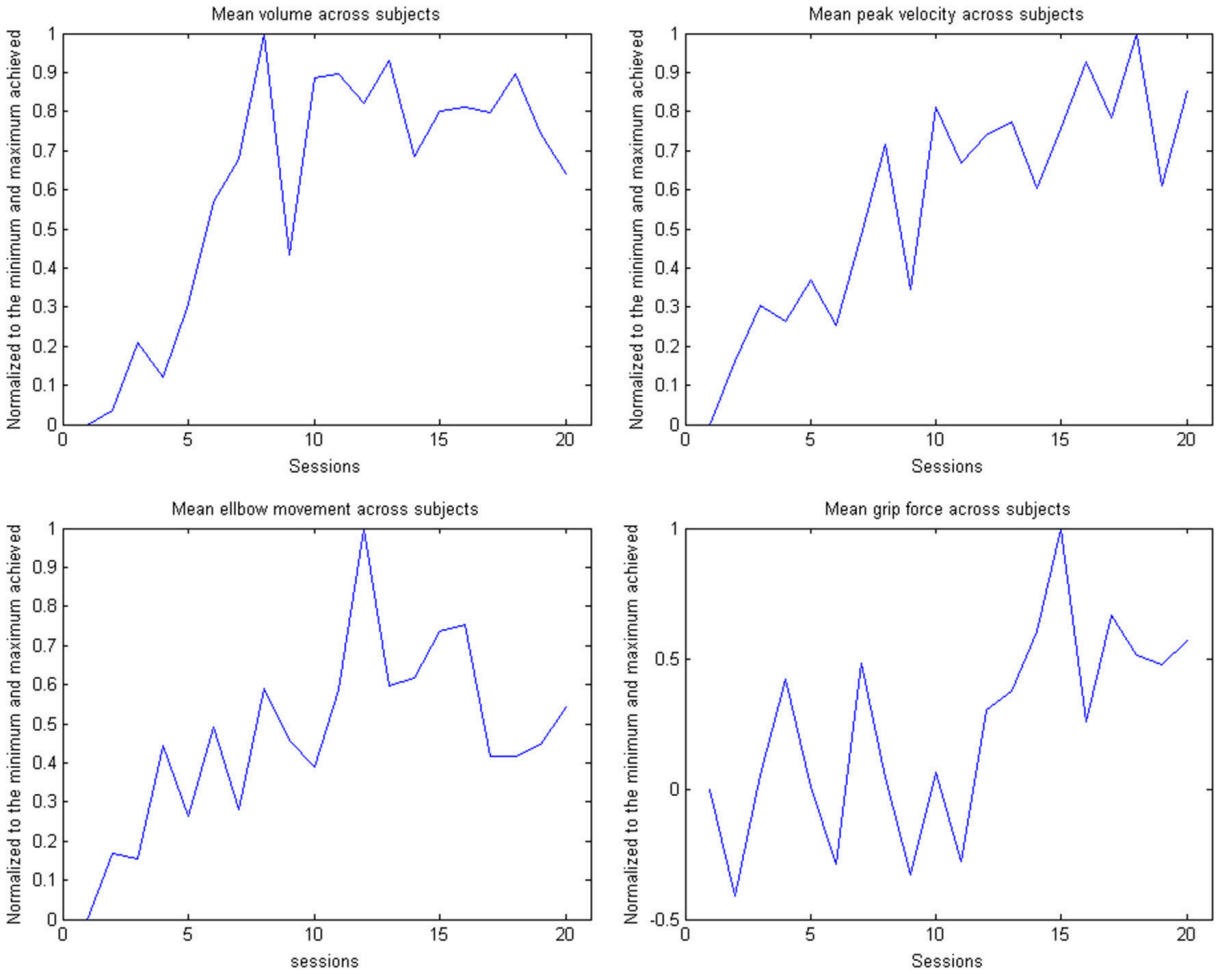
**Evolution of the kinematic parameters volume, peak velocity, elbow movement and mean grip force in the course of the training for the group**. The data is normalized to the maximum performance achieved in each parameter.

Notably, these kinematic gains were also paralleled by significant motor improvements for grip force and elbow movement. The degree of elbow movement increased throughout all sessions by an average of 50% from the 11th to the 16th session, before reaching a saturation level later on. The average grip force also increased relevantly, but showed the largest variability of all the parameters (Figure 6: individual subjects, Figure 7: group data normalized to baseline, Figure 8: group data normalized to maximum). Shoulder movement and upper-arm movement showed an improvement but missed significance; the wrist movement did not change in the course of the training. The UE-FMA score changed significantly (*p* = 0.026) from 14.3 ± 5.4 [from 9 to 22] before to 16.9 ± 6.1 [from 10 to 26] after the intervention.

## Discussion

This pilot study demonstrates the feasibility of progressively increasing the range of motion of chronic stroke patients with a severe impairment of the upper extremity in the course of 20 training sessions. A multi-joint exoskeleton for the paretic arm allowed the subjects to perform task-oriented practice within a virtual environment (Housman et al., 2009). Notably, unlike other studies with similarly affected stroke patients, where active robots completed a movement when started once (Klamroth-Marganska et al., 2014; Brauchle et al., 2015), this assistive technology delivered antigravity-support only and provided no guidance. Patient engagement was maximized by default in the present study, leaving no room for slacking; the continuous visual feedback of the arm kinematics enabled the patients to adjust their action online during each task; an approach that closely resembles natural motor learning.

Such a closed-loop framework follows an operant conditioning rationale. It provides contingent feedback to facilitate the targeted activity which is considered to be beneficial for recovery, and might ultimately lead to functional gains (Gharabaghi et al., 2014c,d; Bauer and Gharabaghi, 2015a). These restorative approaches may, however, pose a considerable challenge for the patients (Bauer and Gharabaghi, 2015b; Fels et al., 2015) who might explore alternative, i.e., therapeutically undesirable, strategies (Gharabaghi et al., 2014b). Moreover, particularly in patients with severe impairments, non-successful trials may cause frustration, thereby limiting motor learning. In this context, closed-loop adaptation of exercise difficulty, as practiced in the present study, may help to avoid frustration by tailoring the range of motion in accordance with the actual ability of each patient.

Previous adaptation approaches provided different types of assistance (Colombo et al., 2012), applied a lead-lag performance model for robotic assistance (Chemuturi et al., 2013), or adjusted the robot/patient's interaction forces (Vergaro et al., 2010). The adaptation approach implemented in this study was differed conceptually from the previous algorithms in that it modulated the virtual task difficulty, not the degree of assistance. This passive gravity compensation remained stable throughout the exercises. Nonetheless, the patients were challenged continuously in our study since the difficulty level increased progressively as soon as task accomplishment was repeated successfully. This performance-dependent online adjustment of task challenge facilitated reinforcement learning and resulted in a progressive increase of the virtual training space with a concurrent improvement of real world range of motion and other kinematic parameters such as accuracy and movement velocity. Notably, these gains followed unsupervised training algorithms and were paralleled by motor improvements such as increased elbow movement, grip force and upper extremity Fugl-Meyer-Assessment score. Whether or not these motor improvements were caused by the specific performance-dependent training algorithm applied here cannot be concluded from the present data, since a control group, i.e., dose-matched training without online adaptation of task difficulty, was not included in this study. Furthermore, this set-up did not assess whether its effects would be limited to chronic and severely affected stroke survivors. The dataset was also small and the heterogeneity of subjects, injuries or time from stroke might influence the gains observed.

However, these limitations do not compromise the major finding of this study, namely the feasibility of progressively increasing the assisted range of motion of severely impaired stroke patients by applying closed-loop virtual reality feedback for unsupervised motor learning. As in all previous studies in chronic stroke patients with severe motor impairments of the upper extremity (e.g., Lang et al., 2016), the clinical improvement within 20 training sessions was, in any case, too modest to lead to relevant functional gains of the patients in their activities of daily living. A sufficiently powered, randomized and adequately controlled but costly trial is, therefore, currently not justified on the basis of this specific approach and the dose of practice applied here. However, the implemented set-up may prove suitable as the basis and training framework for other concurrently applied restorative interventions (see below).

Different, mutually non-exclusive reasons might be responsible for the current limited functional gain: Since the dose of stroke rehabilitation therapy has been shown to correlate positively with clinically meaningful improvements (Lohse et al., 2014; Pollock et al., 2014), the approximately 3000 movement attempts, i.e., exercise trials, performed in the course of 20 sessions during this 4-week study might have been insufficient to induce functionally more relevant improvements. On the other hand, even higher doses of motor therapy (i.e., 6400 or 9600 repetitions in the course of 8 weeks and 32 sessions, 4 days/week) in chronic stroke patients with long-standing (>6 months) upper limb paresis, did not result in a larger functional improvements than in patients who received a therapy dose (3200 repetitions) similar to the one applied here (Lang et al., 2016).

However, the trajectories of kinematic and clinical parameters in the course of the training of the present study suggest that a plateau level of improvement, i.e., a ceiling effect, has not been achieved yet and that further practice sessions, i.e., a longer intervention period, would result in larger gains. Moreover, the huge performance variability of the patients in some sessions, e.g., between 100 and 1000% increased ranges of motion, suggests a general capacity for even larger improvements for at least some of the patients. These *windows of opportunity* might, however, necessitate additional interventions to maximally exploit and consolidate the salvaged restorative potential.

Brain stimulation may facilitate such additive effects for assisted reach-to-grasp exercises: Bilateral transcranial direct current stimulation, for example, has led to improved motor performance of healthy patients beyond the natural learning curve when applied prior to training with the very same multi-joint arm exoskeleton as applied in the present work (Naros et al., 2016a). Brain state-dependent transcranial magnetic stimulation has, moreover, been demonstrated to induce robust increases of corticospinal excitability (Kraus et al., 2016b; Royter and Gharabaghi, 2016) and may thereby amplify use-dependent plasticity when applied in conjunction with assistive rehabilitation devices (Gharabaghi, 2015; Massie et al., 2015). Concurrent state-dependent transcranial magnetic stimulation may thereby unmask latent corticospinal connectivity after stroke (Gharabaghi et al., 2014a) which can be detected and monitored with refined motor mapping techniques (Kraus and Gharabaghi, 2015, 2016; Mathew et al., 2016). Applying phase-dependent stimulation (Raco et al., 2016) synchronized to maximum gains of assisted range of motion, may furthermore consolidate the involved corticospinal circuits in accordance with Hebbian-like plasticity rules.

The scope for recovery may also be improved when using advanced assistive rehabilitation technology based on brain-robot interfaces, since these devices were found to constitute a back-door to the motor system (Gomez-Rodriguez et al., 2011; Bauer et al., 2015). Exercises based on brain-robot feedback of motor-imagery related sensorimotor beta-band desynchronization may result in connectivity changes of cortico-cortical motor networks (Vukelić et al., 2014; Vukelić and Gharabaghi, 2015a,b), lead to a re-distribution of cortico-spinal connections (Kraus et al., 2016a) and to behavioral gains (Naros et al., 2016b). Combining these tools with an adaptive virtual environment similar to that applied in this study may thus maximize the impact of both approaches on sensorimotor function.

In summary, combining gravity-compensation with auto-adaptive closed-loop feedback in virtual reality provides customized rehabilitation environments for severely affected patients and may facilitate unsupervised motor learning by balancing the patient's challenge in accordance with the individual capacity for functional restoration; a proposal that requires investigation in a larger cohort of stroke patients in comparison to sham adaptive and non-adaptive feedback as well as to dose-matched, conventional physiotherapy.

## Author contributions

FG participated in the study design and software development, supervised the measurement sessions and carried out most of the data analysis. GN supervised the measurement sessions. AG participated in the study design and data analysis, and wrote the manuscript. Authors jointly approved the final manuscript.

### Conflict of interest statement

The authors declare that the research was conducted in the absence of any commercial or financial relationships that could be construed as a potential conflict of interest.

## References

[B1] Bauer R. Fels M. Royter V. Raco V. Gharabaghi A. (2016a). Closed-loop adaptation of neurofeedback based on mental effort facilitates reinforcement learning of brain self-regulation. Clin. Neurophysiol. 127, 3156–3164. 10.1016/j.clinph.2016.06.020 27474965

[B2] Bauer R. Fels M. Vukelić M. Ziemann U. Gharabaghi A. (2015). Bridging the gap between motor imagery and motor execution with a brain–robot interface. NeuroImage 108, 319–327. 10.1016/j.neuroimage.2014.12.026 25527239

[B3] Bauer R. Gharabaghi A. (2015a). Reinforcement learning for adaptive threshold control of restorative brain-computer interfaces: a Bayesian simulation. Front. Neurosci. 9:36. 10.3389/fnins.2015.00036 25729347 PMC4325901

[B4] Bauer R. Gharabaghi A. (2015b). Estimating cognitive load during self-regulation of brain activity and neurofeedback with therapeutic brain-computer interfaces. Front. Behav. Neurosci. 9:21. 10.3389/fnbeh.2015.00021 25762908 PMC4329795

[B5] Bauer R. Vukelić M. Gharabaghi A. (2016b). What is the optimal task difficulty for reinforcement learning of brain self-regulation? Clin. Neurophysiol. 127, 3033–3041. 10.1016/j.clinph.2016.06.016 27472538

[B6] Boyd L. A. Winstein C. J. (2004). Providing explicit information disrupts implicit motor learning after basal ganglia stroke. Learn. Mem. 11, 388–396. 10.1101/lm.80104 15286181 PMC498316

[B7] Boyd L. Winstein C. (2006). Explicit information interferes with implicit motor learning of both continuous and discrete movement tasks after stroke. J. Neurol. Phys. Ther. 30, 46–57. 10.1097/01.NPT.0000282566.48050.9b 16796767

[B8] Brauchle D. Vukelić M. Bauer R. Gharabaghi A. (2015). Brain state-dependent robotic reaching movement with a multi-joint arm exoskeleton: combining brain-machine interfacing and robotic rehabilitation. Front. Hum. Neurosci. 9:564. 10.3389/fnhum.2015.00564 26528168 PMC4607784

[B9] Byl N. Abrams G. Pitsch E. Fedulow I. Kim H. Simkins M.. (2013). Chronic stroke survivors achieve comparable outcomes following virtual task specific repetitive training guided by a wearable robotic orthosis (UL-EXO7) and actual task specific repetitive training guided by a physical therapist. J.Hand Ther. 26, 343–351. 10.1016/j.jht.2013.06.001 23911077

[B10] Chase A. (2014). Stroke: improved lesion-symptom mapping in poststroke aphasia. Nat. Rev. Neurol. 10:674. 10.1038/nrneurol.2014.217 25385335

[B11] Chemuturi R. Amirabdollahian F. Dautenhahn K. (2013). Adaptive training algorithm for robot-assisted upper-arm rehabilitation, applicable to individualised and therapeutic human-robot interaction. J. Neuroeng. Rehabil. 10:102. 10.1186/1743-0003-10-102 24073670 PMC3849953

[B12] Cirstea C. M. Ptito A. Levin M. F. (2006). Feedback and cognition in arm motor skill reacquisition after stroke. Stroke 37, 1237–1242. 10.1161/01.STR.0000217417.89347.63 16601218

[B13] Cirstea M. C. Levin M. F. (2000). Compensatory strategies for reaching in stroke. Brain 123 (Pt 5), 940–953. 10.1093/brain/123.5.940 10775539

[B14] Cirstea M. C. Levin M. F. (2007). Improvement of arm movement patterns and endpoint control depends on type of feedback during practice in stroke survivors. Neurorehabil. Neural Repair 21, 398–411. 10.1177/1545968306298414 17369514

[B15] Colombo R. Sterpi I. Mazzone A. Delconte C. Pisano F. (2012). Taking a lesson from patients' recovery strategies to optimize training during robot-aided rehabilitation. IEEE Trans. Neural. Syst. Rehabil. Eng. 20, 276–285. 10.1109/TNSRE.2012.2195679 22623406

[B16] Dobkin B. H. (2005). Rehabilitation after stroke. New Eng. J. Med. 352, 1677–1684. 10.1056/NEJMcp043511 15843670 PMC4106469

[B17] Fels M. Bauer R. Gharabaghi A. (2015). Predicting workload profiles of brain–robot interface and electromygraphic neurofeedback with cortical resting-state networks: personal trait or task-specific challenge? J. Neural Eng. 12:046029. 10.1088/1741-2560/12/4/046029 26170164

[B18] Gharabaghi A. (2015). Activity-dependent brain stimulation and robot-assisted movements for use-dependent plasticity. Clin. Neurophysiol. 126, 853–854. 10.1016/j.clinph.2014.09.004 25260322

[B19] Gharabaghi A. Kraus D. Leão M. T. Spüler M. Walter A. Bogdan M.. (2014a). Coupling brain-machine interfaces with cortical stimulation for brain-state dependent stimulation: enhancing motor cortex excitability for neurorehabilitation. Front. Hum. Neurosci. 8:122. 10.3389/fnhum.2014.00122 24634650 PMC3942791

[B20] Gharabaghi A. Naros G. Khademi F. Jesser J. Spüler M. Walter A.. (2014b). Learned self-regulation of the lesioned brain with epidural electrocorticography. Front. Behav. Neurosci. 8:429. 10.3389/fnbeh.2014.00429 25538591 PMC4260503

[B21] Gharabaghi A. Naros G. Walter A. Grimm F. Schuermeyer M. Roth A. (2014c). From assistance towards restoration with an implanted brain–computer interface based on epidural electrocorticography: a single case study. Restor. Neurol. Neurosci. 32, 517–525. 10.1016/j.clinph.2016.06.016 25015699

[B22] Gharabaghi A. Naros G. Walter A. Roth A. Bogdan M. Rosenstiel W.. (2014d). Epidural electrocorticography of phantom hand movement following long-term upper-limb amputation. Front. Hum. Neurosci. 8:285. 10.3389/fnhum.2014.00285 24834047 PMC4018546

[B23] Gomez-Rodriguez M. Peters J. Hill J. Schölkopf B. Gharabaghi A. Grosse-Wentrup M. (2011). Closing the sensorimotor loop: haptic feedback facilitates decoding of motor imagery. J. Neural Eng. 8:036005. 10.1088/1741-2560/8/3/036005 21474878

[B24] Grimm F. Gharabaghi A. (2016). Closed-loop neuroprosthesis for reach-to-grasp assistance: combining adaptive multi-channel neuromuscular stimulation with a multi-joint arm exoskeleton. Front. Neurosci. 10:284. 10.3389/fnins.2016.00284 27445658 PMC4917563

[B25] Grimm F. Naros G. Gharabaghi A. (2016a). Compensation or restoration: closed-loop feedback of movement quality for assisted reach-to-grasp exercises with a multi-joint arm exoskeleton. Front. Neurosci. 10:280. 10.3389/fnins.2016.00280 27445655 PMC4914560

[B26] Grimm F. Walter A. Spüler M. Naros G. Rosenstiel W. Gharabaghi A. (2016b). Hybrid neuroprosthesis for the upper limb: combining brain-controlled neuromuscular stimulation with a multi-joint arm exoskeleton. Front. Neurosci. 10:367. 10.3389/fnins.2016.00367 27555805 PMC4977295

[B27] Holland P. W. Welsch R. E. (1977). Robust regression using iteratively reweighted least-squares. Commun. Stat. Theory Methods 6, 813–827. 10.1080/03610927708827533

[B28] Housman S. J. Scott K. M. Reinkensmeyer D. J. (2009). A randomized controlled trial of gravity-supported, computer-enhanced arm exercise for individuals with severe hemiparesis. Neurorehabil. Neural Repair 23, 505–514. 10.1177/1545968308331148 19237734

[B29] Jørgensen H. S. Nakayama H. Raaschou H. O. Olsen T. S. (1999). Stroke. neurologic and functional recovery the copenhagen stroke study. Phys. Med. Rehabil. Clin. N. Am. 10, 887–906. 10573714

[B30] Kitago T. Goldsmith J. Harran M. Kane L. Berard J. Huang S.. (2015). Robotic therapy for chronic stroke: general recovery of impairment or improved task-specific skill? J. Neurophysiol. 114, 1885–1894. 10.1152/jn.00336.2015 26180120 PMC4575974

[B31] Kitago T. Liang J. Huang V. S. Hayes S. Simon P. Tenteromano L.. (2013). Improvement after constraint-induced movement therapy: recovery of normal motor control or task-specific compensation? Neurorehabil. Neural Repair 27, 99–109. 10.1016/j.clinph.2016.06.016 22798152

[B32] Klamroth-Marganska V. Blanco J. Campen K. Curt A. Dietz V. Ettlin T.. (2014). Three-dimensional, task-specific robot therapy of the arm after stroke: a multicentre, parallel-group randomised trial. Lancet Neurol. 13, 159–166. 10.1016/S1474-4422(13)70305-3 24382580

[B33] Kraus D. Gharabaghi A. (2015). Projecting navigated TMS sites on the gyral anatomy decreases inter-subject variability of cortical motor maps. Brain Stimul. 8, 831–837. 10.1016/j.brs.2015.03.006 25865772

[B34] Kraus D. Gharabaghi A. (2016). Neuromuscular plasticity: disentangling stable and variable motor maps in the human sensorimotor cortex. Neural Plastic. 2016:7365609. 10.1155/2016/7365609 27610248 PMC5004060

[B35] Kraus D. Naros G. Bauer R. Khademi F. Leão M. T. Ziemann U.. (2016b). Brain state-dependent transcranial magnetic closed-loop stimulation controlled by sensorimotor desynchronization induces robust increase of corticospinal excitability. Brain Stimul. 9, 415–424. 10.1016/j.brs.2016.02.007 26970878

[B36] Kraus D. Naros G. Bauer R. Leão M. T. Ziemann U. Gharabaghi A. (2016a). Brain–robot interface driven plasticity: Distributed modulation of corticospinal excitability. NeuroImage 125, 522–532. 10.1016/j.neuroimage.2015.09.074 26505298

[B37] Kwakkel G. Meskers C. G. M. (2014). Effects of robotic therapy of the arm after stroke. Lancet Neurol. 13, 132–133. 10.1016/S1474-4422(13)70285-0 24382581

[B38] Lang C. E. Strube M. J. Bland M. D. Waddell K. J. Cherry-Allen K. M. Nudo R. J.. (2016). Dose response of task-specific upper limb training in people at least 6 months poststroke: a phase II, single-blind, randomized, controlled trial. Ann. Neurol. 80, 342–354. 10.1002/ana.24734 27447365 PMC5016233

[B39] Laver K. E. George S. Thomas S. Deutsch J. E. Crotty M. (2015). Virtual reality for stroke rehabilitation. Cochrane Database Syst. Rev. 2:CD008349 10.1002/14651858.CD008349.pub2 PMC6465102 25927099

[B40] Lo A. C. Guarino P. D. Richards L. G. Haselkorn J. K. Wittenberg G. F. Federman D. G.. (2010). Robot-assisted therapy for long-term upper-limb impairment after stroke. New Eng. J. Med. 362, 1772–1783. 10.1056/NEJMoa0911341 20400552 PMC5592692

[B41] Lohse K. R. Lang C. E. Boyd L. A. (2014). Is more better? Using metadata to explore dose-response relationships in stroke rehabilitation. Stroke 45, 2053–2058. 10.1161/strokeaha.114.004695 24867924 PMC4071164

[B42] Massie C. L. Kantak S. S. Narayanan P. Wittenberg G. F. (2015). Timing of motor cortical stimulation during planar robotic training differentially impacts neuroplasticity in older adults. Clin. Neurophysiol. 126, 1024–1032. 10.1016/j.clinph.2014.06.053 25283712 PMC4362917

[B43] Mathew J. Kübler A. Bauer R. Gharabaghi A. (2016). Probing corticospinal recruitment patterns and functional synergies with transcranial magnetic stimulation. Front. Cell. Neurosci. 5:175. 10.3389/fncel.2016.00175 27458344 PMC4932869

[B44] Mehrholz J. Pohl M. Platz T. Kugler J. Elsner B. (2015). Electromechanical and robot-assisted arm training for improving activities of daily living, armfunction, and arm muscle strength after stroke. Cochrane Database Syst. Rev. 11:CD006876. 10.1002/14651858.CD006876.pub3 26559225 PMC6465047

[B45] Metzger J. C. Lambercy O. Califfi A. Dinacci D. Petrillo C. Rossi P.. (2014). Assessment-driven selection and adaptation of exercise difficulty in robot-assisted therapy: a pilot study with a hand rehabilitation robot. J. Neuroeng. Rehabil. 11:154. 10.1186/1743-0003-11-154 25399249 PMC4273449

[B46] Naros G. Geyer M. Koch S. Mayr L. Ellinger T. Grimm F.. (2016a). Enhanced motor learning with bilateral transcranial direct current stimulation: impact of polarity or current flow direction? Clin. Neurophysiol. 127, 2119–2126. 10.1016/j.clinph.2015.12.020 26818881

[B47] Naros G. Gharabaghi A. (2015). Reinforcement learning of self-regulated β-oscillations for motor restoration in chronic stroke. Front. Hum. Neurosci. 9:391. 10.3389/fnhum.2015.00391 26190995 PMC4490244

[B48] Naros G. Naros I. Grimm F. Ziemann U. Gharabaghi A. (2016b). Reinforcement learning of self-regulated sensorimotor β-oscillations improves motor performance. Neuroimage 134, 142–152. 10.1016/j.neuroimage.2016.03.016 27046109

[B49] Pollock A. Farmer S. E. Brady M. C. Langhorne P. Mead G. E. Mehrholz J. (2014). Interventions for improving upper limb function after stroke. Cochrane Database Syst. Rev. 11:CD010820 10.1002/14651858.cd010820.pub2 PMC6469541 25387001

[B50] Raco V. Bauer R. Tharsan S. Gharabaghi A. (2016). Combining TMS and tACS for closed-loop phase-dependent modulation of corticospinal excitability: a feasibility study. Front. Cell. Neurosci. 10:143. 10.3389/fncel.2016.00143 27252625 PMC4879130

[B51] Reis J. Schambra H. M. Cohen L. G. Buch E. R. Fritsch B. Zarahn E.. (2009). Noninvasive cortical stimulation enhances motor skill acquisition over multiple days through an effect on consolidation. Proc. Natl. Acad. Sci. U.S.A. 106, 1590–1595. 10.1016/j.clinph.2016.06.016 19164589 PMC2635787

[B52] Royter V. Gharabaghi A. (2016). Brain state-dependent closed-loop modulation of paired associative stimulation controlled by sensorimotor desynchronization. Front. Cell. Neurosci. 10:115. 10.3389/fncel.2016.00115 27242429 PMC4861730

[B53] Shmuelof L. Krakauer J. W. Mazzoni P. (2012). How is a motor skill learned? Change and invariance at the levels of task success and trajectory control. J. Neurophysiol. 108, 578–594. 10.1016/j.clinph.2016.06.016 22514286 PMC3404800

[B54] Subramanian S. K. Lourenço C. B. Chilingaryan G. Sveistrup H. Levin M. F. (2013). Arm motor recovery using a virtual reality intervention in chronic stroke: randomized control trial. Neurorehabil. Neural Repair 27, 13–23. 10.1177/1545968312449695 22785001

[B55] Vergaro E. Casadio M. Squeri V. Giannoni P. Morasso P. Sanguineti V. (2010). Self-adaptive robot training of stroke survivors for continuous tracking movements. J. Neuroeng. Rehabil. 7:13. 10.1186/1743-0003-7-13 20230610 PMC2850909

[B56] Vukelić M. Bauer R. Naros G. Naros I. Braun C. Gharabaghi A. (2014). Lateralized alpha-band cortical networks regulate volitional modulation of beta-band sensorimotor oscillations. NeuroImage 87, 147–153. 10.1016/j.neuroimage.2013.10.003 24121086

[B57] Vukelić M. Gharabaghi A. (2015a). Oscillatory entrainment of the motor cortical network during motor imagery is modulated by the feedback modality. Neuroimage 111, 1–11. 10.1016/j.neuroimage.2015.01.058 25665968

[B58] Vukelić M. Gharabaghi A. (2015b). Self-regulation of circumscribed brain activity modulates spatially selective and frequency specific connectivity of distributed resting state networks. Front. Behav. Neurosci. 9:181. 10.3389/fnbeh.2015.00181 26236207 PMC4500921

[B59] Wittmann F. Lambercy O. Gonzenbach R. R. van Raai M. A. Hover R. Held J. (2015). Assessment-driven arm therapy at home using an IMU-based virtual reality system. IEEE Rehabil. Robot. (ICORR) 707–712. 10.1109/ICORR.2015.7281284

